# Beliefs about willpower moderate the effect of previous day demands on next day’s expectations and effective goal striving

**DOI:** 10.3389/fpsyg.2015.01496

**Published:** 2015-10-14

**Authors:** Katharina Bernecker, Veronika Job

**Affiliations:** ^1^Psychology of Motivation, Volition, and Emotion, Department of Psychology, University of ZurichZurich, Switzerland; ^2^Developmental Psychology: Adulthood, Department of Psychology, University of ZurichZurich, Switzerland

**Keywords:** implicit theories about willpower, goal striving, self-control, optimism, self-efficacy

## Abstract

Research suggests that beliefs about willpower affect self-regulation following previous self-regulatory demands (Job et al., 2010). Some people believe that their willpower is limited, meaning that after a demanding task it needs to be replenished (limited theory). By contrast, others believe that willpower is not limited and that previous self-control tasks even activate willpower (non-limited theory). We hypothesized that when people experience a demanding day their beliefs about willpower predict their expected capacity to self-regulate and their actual self-regulation on the following day. In a daily diary study (*N* = 157), we measured students’ daily level of demands, their expected performance in unpleasant tasks, and their effective goal striving. Results showed that following a demanding day, students with a non-limited theory had higher expectations about their progress in unpleasant tasks and were striving more efficiently for their goals than students with a limited theory. These findings suggest that beliefs about willpower affect whether demands experienced on a previous day have positive or negative consequences on people’s self-regulation.

## Introduction

Over a century ago, William James (1907) argued that people’s levels of physical and mental energy are not always the same but change from day to day. He argued that “Every one is familiar with the phenomenon of feeling more or less alive on different days. Every one knows on any given day that there are energies slumbering in him which the incitements of that day do not call forth, but which he might display if these were greater” (p. 322). In recent years, the idea of temporal changes in energy levels was applied to an important mental capacity, namely self-control. Self-control is the ability to alter thoughts, emotions, and behaviors in a way that helps individuals to achieve their long-term goals (Baumeister et al., 1998, 2007). The influential strength model of self-control argues that self-control fluctuates because it is based on a limited resource, which gets easily depleted when self-control is exerted (Baumeister and Heatherton, 1996; Baumeister, 2002; Oaten and Cheng, 2005). As support for the model numerous experimental studies found that self-control performance is impaired by previous acts of self-control (Hagger et al., 2010). Thus, according to the strength model, self-control capacity fluctuates due to the depletion of a limited resource.

James (1907) had a different idea about what might cause fluctuations in daily levels of energy. In contrast to the strength model of self-control, he argued that people generally possess an indefinite amount of mental and physical resources; what changes is how much they tap into these resources on a given day. He further believed that most people habitually fail to use the plenty reservoirs of energy they possess (James, 1907). Recent empirical findings support this notion and suggest that people’s beliefs about willpower affect self-control performance following initial self-control exertion (Job et al., 2010). People with a limited theory believe that exerting self-control, for instance, by working on a strenuous mental task or resisting a temptation, depletes their willpower resource. In order to be available again the resource needs to be restored, for instance, by taking a break or eating. In contrast, people with a non-limited theory believe that exerting self-control can even activate their willpower (Job et al., 2010). Several laboratory experiments showed that people who endorse a limited theory show self-control impairments after initial self-control exertion, while people with a non-limited theory perform well regardless of previous self-control demands (Job et al., 2010; Miller et al., 2012). Importantly, the same pattern of results emerged when willpower theories were not measured but manipulated (Job et al., 2010; Miller et al., 2012). These results suggest that willpower beliefs play a causal role and determine whether people are able to recruit the required willpower to succeed in consecutive self-control tasks.

Despite these apparently functional effects of a non-limited theory, a recent study suggests that a non-limited theory may backfire when people face sustained self-regulatory demands (Vohs et al., 2013). Whereas participants with an induced non-limited theory outperformed participants with an induced limited theory when self-regulatory performance was measured after mild depletion (two self-control tasks), the pattern reversed after severe depletion (four self-control tasks) (Vohs et al., 2013). Based on these findings it was speculated that a non-limited theory may persuade people to overexert their resources, resulting in self-control failure in the face of high demands. In spite of these considerations, field studies examining self-control performance in real life show that people with a non-limited theory are even better able to exert self-control in phases of high self-regulatory demands than people with a limited theory (Job et al., 2010, 2015b). In one study, students were surveyed once at the beginning, once in the middle, and once at the end of the term during final exams (Job et al., 2010, Study 4). Results showed that in the final exam period students with a limited theory reported more self-regulatory failure than students with a non-limited theory. They procrastinated more, ate less healthy, and reported worse self-regulation with regard to a challenging personal goal. In the middle of the term, when students faced low self-regulatory demands, self-control failure was overall less likely independent of students’ willpower theories (Job et al., 2010, Study 4). In a second study, students were surveyed weekly in the five weeks before their exams (Job et al., 2015b). As an improvement of the previous study, self-regulatory demands (e.g., assignments, interpersonal conflicts, health problems) were assessed alongside with different indicators of self-regulation. Inconsistent with Vohs et al.’s (2013) findings, students with a limited theory reported worse self-regulation when they faced high demands as compared to the average student. In contrast, students with a non-limited theory even showed the opposite trend and reported a healthier diet when facing high demands. This study further assessed students’ grade point average and found that a limited theory predicted lower grades (controlling for previous grades), especially among students with a high course load. Students with a non-limited theory even performed slightly better when they had a heavy course load. For them increased self-regulatory demands had a positive effect on their performance (Job et al., 2015b). In sum, the findings suggest that willpower theories predict self-control performance when demands accumulate and that people with a non-limited theory might even benefit from increased self-regulatory demands.

However, one limitation of both studies is that effects of demands were observed only on the between-subjects level. That is, the effects of willpower theories were compared for individuals with high versus low demands (i.e., comparing individuals’ weekly demands to the average demand of all students in that week, Job et al., 2015b). But willpower theories might also interact with demands on a within-subject level in predicting self-control performance (i.e., comparing weekly demands to individuals’ own average in demands). In one study, within-person effects of demands were examined but turned out to be not significant (Job et al., 2015b). This was probably due to the long time periods between measurement points (i.e., one week), causing the variance in demands to be greater between participants than within participants. Yet, examining within-subject processes is important, because research shows that they do not necessarily converge with between-person processes (Hoffman and Stawski, 2009). Further, between-subjects effects can be caused by third variables or general person characteristics that may explain the differences between subgroups of people in a sample.

The present research, therefore, aims to test whether willpower theories interact with within-person variations in previous demands to predict effective goal striving as a measure for successful self-regulation in everyday life. We hypothesized that people with a limited willpower theory show less effective goal striving when previous demands exceed their own personal average. In contrast, people with a non-limited theory should show effective goal striving even when previous demands exceed their personal average. This is because individuals with a non-limited theory should perceive themselves as having sufficient resources to strive for important personal goals even if they had to deal with previous self-regulatory demands. People endorsing a limited theory should be more guided by the motive to conserve resources once these resources have been taxed and thus apply less of their resources to current personal goals.

Adopting James (1907) idea of daily fluctuations in the use of energy resources we conducted a daily diary study. This design allowed us to test whether demands experienced on any given day affect how effectively people strive for their goals on the next day. The daily diary method allows people to closely discriminate between days and therefore naturally increases the ratio of within- to between-person variance. Further, the high frequency measurement increases the power of detecting a probably small carryover effect of previous day demands to the next day. One reason why we expected previous day demands to matter is that research showed that willpower theories moderate the effects of previous self-regulatory demands (Job et al., 2010). Further, retrospective reports of demands and effective goals striving reported on the same day might influence each other. Therefore, testing effects of the previous day has the methodological advantage of independent measurement of demands and self-control performance.

The second goal of the present study is to examine the role of expectations in the context of willpower theories. When people view their willpower either as limited or non-limited this should affect their expectations about their ability to exert self-control, particularly when their willpower was previously taxed. Individuals who believe that their resources are diminished due to a previous self-control act, might be more motivated to conserve what is left and thus align their expectations about their future capacity to self-control accordingly. Conversely, people who perceive themselves as having sufficient resources should have higher expectations about their future capacity to self-control. These differences in expectations might be one plausible mediator of the effect of willpower theories on self-control performance. However, so far, no study has investigated the effects of willpower theories on expectations. In the present study, we explored whether willpower theories determine individuals’ expectations particularly after a demanding day. Because change on the expectations should be specific to tasks that require self-control, we assessed people’s expectations about their performance in unpleasant and pleasant tasks. Tasks that are unpleasant and do not have any hedonic value should require self-control to perform, because they are at odds with a person’s short-term motives (Fujita, 2011). In contrast, pleasant tasks should not require self-control because they satisfy a person’s short-term motives. Thus, we expected that, after a demanding day (more than after a non-demanding day), willpower theories should affect people’s expected performance in unpleasant tasks but not in pleasant tasks. Further, these differences in expectation might be part of the mechanism and explain why willpower theories interact with demands to predict effective goal striving.

In addition to these task-specific expectations about performance, we also aimed to examine how willpower theories relate to more global expectations, such as optimism, pessimism, and general self-efficacy. Although these constructs should be related to willpower theories (people with a limited theory might be less optimistic, more pessimistic, and have lower general self-efficacy), controlling for these global expectations should not affect the proposed interactive effects of implicit theories about willpower and previous day demands on effective goal striving.

## Materials and Methods

This study was carried out in accordance with the ethical standards of the institutional research committee and with the 1964 Helsinki declaration and its later amendments for comparable ethical standards. Informed consent was obtained from all individual participants included in the study.

### Participants and Procedure

Participants were 157 students from a public university in Switzerland (132 women; *M*_age_ = 22.96 years, Range: 18–51 years) who were recruited via lectures, flyers on campus, mailing lists, and online forums for students.

After signing up for the “Smartphone Study on Well-being” via email participants received a link to the baseline survey in the third week of the spring term. The baseline survey assessed individual difference measures, such as willpower theories, optimism, pessimism, and general self-efficacy. Within the following 9 weeks, participants completed two diary phases of five consecutive days each (Monday to Friday). To ensure sufficient variance in daily demands we placed the first diary phase in the beginning of the term (6^th^ of 15 weeks) and the second diary phase close to the exam period at the end of the term (13^th^ week). On each day participants were emailed a link to a morning survey at 7:00 am with a request to respond until 11:00 am, and a link to an evening survey at 6:00 pm with the request to answer until 11:00 pm. Both links expired at the announced time.

Participants received 10 Swiss Francs ($10.70) for completing the baseline questionnaire, 10 Swiss Francs for completing each diary period and another 20 Swiss Francs for completing 80% of the daily questionnaires. On average, participants completed 18.00 (*SD* = 3.29, Range: 3–20) daily questionnaires. Overall, 1378 out of 1570 (87.8%) morning questionnaires and 1390 out of 1570 (88.5%) evening questionnaires were completed.

### Individual Difference Measures Assessed at Baseline

#### Implicit Theories About Willpower

At baseline, participants completed 12 items assessing implicit theories about willpower with respect to strenuous mental activities and resisting temptations (Job et al., 2010). Example items are “After a strenuous mental activity your energy is depleted and you must rest to get it refueled again” (limited theory) and “Your mental stamina fuels itself; even after strenuous mental exertion you can continue doing more of it” (non-limited theory), which were answered on a 6-point scale (*1 = Strongly agree*, *6 = Strongly disagree*, α = 0.81). Items representing a limited theory were reverse-scored so that on the averaged scale higher values represent greater agreement with a limited theory.

#### General Optimism and Pessimism

To examine whether the effects of implicit theories about willpower were independent of optimism and pessimism, we administered the German version of the revised Life Orientation Test (LOT-R; Herzberg et al., 2006; Glaesmer et al., 2008) at baseline. Participants indicated on a 5-point scale (*1 = Strongly disagree, 5 = Strongly agree*) how much they agreed with three items assessing optimism (e.g., “If something can go wrong for me, it will,” α = 0.68) and three items assessing pessimism (e.g., “In uncertain times, I usually expect the best,” α = 0.62). Because optimism and pessimism represent two independent constructs the items were averaged to two separate scales (Herzberg et al., 2006).

#### General Self-efficacy

We assessed general self-efficacy with the German version of the General Self-efficacy Scale (Schwarzer et al., 1997). Participants answered 10 items (e.g., “I can always manage to solve difficult problems if I try hard enough”) on a 4-point scale (*1* = *Not at all true*, *4* = *Exactly true*, α = *0.77*).

### Daily Measures of Expectations, Demands, and Effective Goal Striving

In the morning questionnaire, we assessed expectations about progress and exhaustion from unpleasant and pleasant tasks. In the evening questionnaire, we assessed daily demands and effective goal striving.

#### Expectations

In the morning, participants were instructed to think about all unpleasant tasks they were facing that day. One item assessed the *expected amount* of unpleasant tasks (“On how many unpleasant tasks do you have to work today?”; *1* = *None*, *5* = *A great many*). Another item assessed their *expected progress* on these unpleasant tasks (“What do you think, how much progress will you make on these tasks?”, *1* = *None*, *5* = *Very much*). Another item assessed their *expected exhaustion* from these unpleasant tasks (“What do you think, how exhausting will it be to work on these tasks?”, *1* = *Not at all*, *5* = *Very much*). As control measure, we let participants answer the same three items about upcoming pleasant tasks of the day. We did not expect any effect of willpower theories on expectations about pleasant tasks.

#### Demands

In the evening, one item assessed demands throughout the day (i.e., “Overall, how demanding was your day?”, 1 = *Not at all*, 5 = *Very much*).

#### Effective Goal Striving

Two items assessed effective goal striving throughout the day (i.e., “Overall, how efficiently have you worked today?”, 1 = *Not at all*, 5 = *Very much;* “How often did you work on things that are important to you? 1 = *All the time*, 6 = *At no time*, 0.56 < α*_day_* < 0.71).

#### Control Variables at Day-level

The order of the days within the diary was coded to control for linear trends over time (*0* = *1^st^ day*, *9* = *10^th^ day*). Further, we controlled in which diary phase the daily survey was completed (*0* = *1^st^ week*, *1* = *2^nd^ week*).

## Results

Data was analyzed with a hierarchical linear modeling approach (Bryk and Raudenbush, 1992) using R (R Core Team, 2013) and the lme4 package (Bates et al., 2015). All day-level variables (Level 1; e.g., demands) were centered at the person mean (cf. Judge et al., 2006), and person-level variables (Level 2; e.g., implicit theories about willpower) at the grand mean. Because day-level variables were centered at the person mean the results do not refer to differences between participants but to daily fluctuations within participants. All models were fitted using a maximum likelihood estimation procedure.

### Preliminary and Descriptive Analyses

For each day-level variable we estimated the *ICC1*, which gives the proportion of between-person variance to the total variance. The values ranged from 0.15 to 0.30, which means that 15–30% of the variance was between person variance. This result suggests that data are dependent on the person and, therefore, justify the use of a multilevel model. However, a substantial portion of the variance in expectations and effective goal striving was within-person, which might be predicted by cross-level interactions between variables measured at day-level (e.g., demands) and person variables (e.g., willpower theory).

**Table 1** shows the descriptive statistics and zero-order correlations between the main variables of the study. Willpower theories were moderately correlated with people’s mean reports on demands and expectations on the upcoming day. People with a limited theory expected a greater amount of unpleasant tasks, less progress, but more exhaustion resulting from working on them on the following day. Furthermore, people with a limited theory, indeed, reported lower effective goal striving. Last, willpower theories were only moderately correlated with optimism, pessimism, and self-efficacy, suggesting that willpower theories differ from these general expectations. As expected, people with a limited theory were slightly less optimistic, more pessimistic, and had lower self-efficacy.

**Table 1:** Descriptive statistics and zero-order correlations between main variables.

	Variable	*M (SD)*	1	2	3	4	5	6	7	8	
(1)	Willpower theory	3.34 (0.47)									
(2)	Demands	2.30 (1.04)	0.10		0.18	0.00	0.15	0.15			
(3)	Expected amount of UT	2.48 (0.87)	0.17	0.36		-0.15	0.48	0.05			
(4)	Expected progress in UT	3.65 (0.88)	-0.20	-0.07	-0.21		-0.23	0.23			
(5)	Expected exhaustion from UT	3.37 (0.96)	0.12	0.19	0.48	-0.26		0.09			
(6)	Effective goal striving	3.05 (1.21)	-0.16	0.15	-0.09	0.27	0.04				
(7)	Optimism	3.68 (0.75)	-0.15	-0.01	-0.14	0.04	-0.09	0.20			
(8)	Pessimism	2.30 (0.73)	0.11	0.13	0.16	0.01	-0.04	0.01	-0.40		
(9)	Self-efficacy	2.87 (0.38)	-0.23	-0.03	-0.25	0.21	-0.14	0.17	0.36	-0.25	

*Correlations below the diagonal are person-level correlations (N = 157) with correlations r > | 0.16| being significant at p < 0.05. Correlations above the diagonal are day-level correlations (N = 1390) with correlations r > | 0.05| being significant at p < 0.05. UT, unpleasant tasks.*

### Hypothesis Tests

#### Do People with a Limited Theory Experience More Demands at Day-level?

No. We predicted daily demands by implicit theories about willpower controlling for the effects of day and week. As expected, demands were perceived to be higher in the second diary week, *b* = 0.34, *SE* = 0.12, *t*(1093) = 2.76, *p* = 0.006 (0.09, 0.58), but there was no linear trend over time, *b* = -0.02, *SE* = 0.02, *t*(1093) = -1.20, *p* = 0.228 (-0.06, 0.02). As expected, there was no effect of implicit theories about willpower on demands, *b* = 0.13, *SE* = 0.10, *t*(152) = 1.33, *p* = 0.185 (-0.06, 0.32). This finding replicates previous research showing that willpower theories are not related to people’s demands (Job et al., 2015b). The independence of the two constructs also allows testing effects of their two-way interaction on expectations and effective goal striving.

#### Do People with a Limited Theory Expect to Have More Unpleasant Tasks, Particularly After a Demanding Day?

No. We analyzed the effects of willpower theories on expectations about the amount of unpleasant and pleasant tasks separately. In each model, the main predictors were willpower theories, previous day demands, and their two-way interaction. Additionally, we controlled for effects of day, week, person mean demands, same day demands, optimism, pessimism, and self-efficacy.

In the first model, we predicted the expected amount of unpleasant tasks. The random effect of previous day demands was added, because it significantly improved the model fit, which was tested with a likelihood ratio test, *X*^2^(2) = 12.10, *p* = 0.002. Results are summarized in **Table 2**. The effect of willpower theories on the amount of unpleasant tasks was not significant, while the effect of previous day demands was significant. Demands of the previous day increased the expected amount of unpleasant tasks reported the next morning. The interaction between willpower theories and previous day demands was not significant. We ran the same set of analysis on the expected amount of pleasant tasks, but the main effect of willpower theory and previous day demands, as well as their interaction were not significant, *t*s < 1. As expected, willpower theories did not affect the expectations about the amount of unpleasant or pleasant tasks.

**Table 2:** Linear multilevel model predicting expected amount of, progress in, and exhaustion from unpleasant tasks.

		Expected amount^a^					Expected progress^b^					Expected exhaustion^c^			
									
				95% CI					95% CI					95% CI	
			
Variable	*b*	*SE*	*p*	lower	upper	*b*	*SE*	*p*	lower	upper	*b*	*SE*	*p*	lower	upper
Intercept	1.45	0.19	0.000	1.07	1.84	3.82	0.19	0.000	3.45	4.20	2.79	0.20	0.000	2.41	3.17
Day	0.02	0.02	0.249	-0.02	0.07	-0.04	0.02	0.088	-0.09	0.01	-0.04	0.03	0.100	-0.09	0.01
Week	0.32	0.12	0.007	0.09	0.55	0.09	0.14	0.503	-0.17	0.36	0.48	0.14	0.001	0.19	0.76
Optimism	-0.06	0.07	0.355	-0.19	0.07	-0.03	0.06	0.677	-0.15	0.10	-0.04	0.06	0.538	-0.17	0.09
Pessimism	0.03	0.07	0.707	-0.11	0.16	-0.02	0.07	0.766	-0.15	0.11	-0.08	0.07	0.220	-0.21	0.05
Self-efficacy	-0.19	0.13	0.139	-0.44	0.06	0.26	0.12	0.037	0.02	0.51	-0.08	0.12	0.536	-0.32	0.17
Person mean demands	0.31	0.08	0.000	0.16	0.46	-0.02	0.08	0.839	-0.16	0.13	0.24	0.08	0.002	0.10	0.39
Same day demands	0.03	0.03	0.358	-0.03	0.08	0.02	0.03	0.581	-0.04	0.08	0.08	0.03	0.016	0.02	0.15
Previous day demands	0.07	0.03	0.030	0.01	0.13	0.04	0.03	0.167	-0.02	0.11	0.06	0.03	0.108	-0.01	0.12
Willpower theory	0.09	0.10	0.320	-0.09	0.28	-0.13	0.09	0.161	-0.32	0.05	0.08	0.09	0.392	-0.10	0.26
Willpower theory^∗^															
Previous day demands	0.09	0.06	0.140	-0.03	0.21	-0.13	0.06	0.027	-0.25	-0.02	0.16	0.06	0.012	0.04	0.29
Variance components															
Intercept (*SD*)	0.38			0.29	0.49	0.38			0.31	0.47	0.22			0.10	0.52
Demands (*SD*)	0.15			0.08	0.27	-			-	-	-			-	-
Residual (*SD*)	0.72			0.67	0.77	0.77			0.72	0.81	0.89			0.83	0.95
Correlation	-0.27			-0.74	0.37	-			-	-	-			-	-

*^a^df_within_ = 145, df_between_ = 687; ^b^df_within_ = 145, df_between_ = 669; ^c^df_within_ = 145, df_between_ = 674.*

#### Do People with a Limited Theory Expect to Make Less Progress in Unpleasant Tasks, Particularly After a Demanding Day?

**FIGURE 1:**
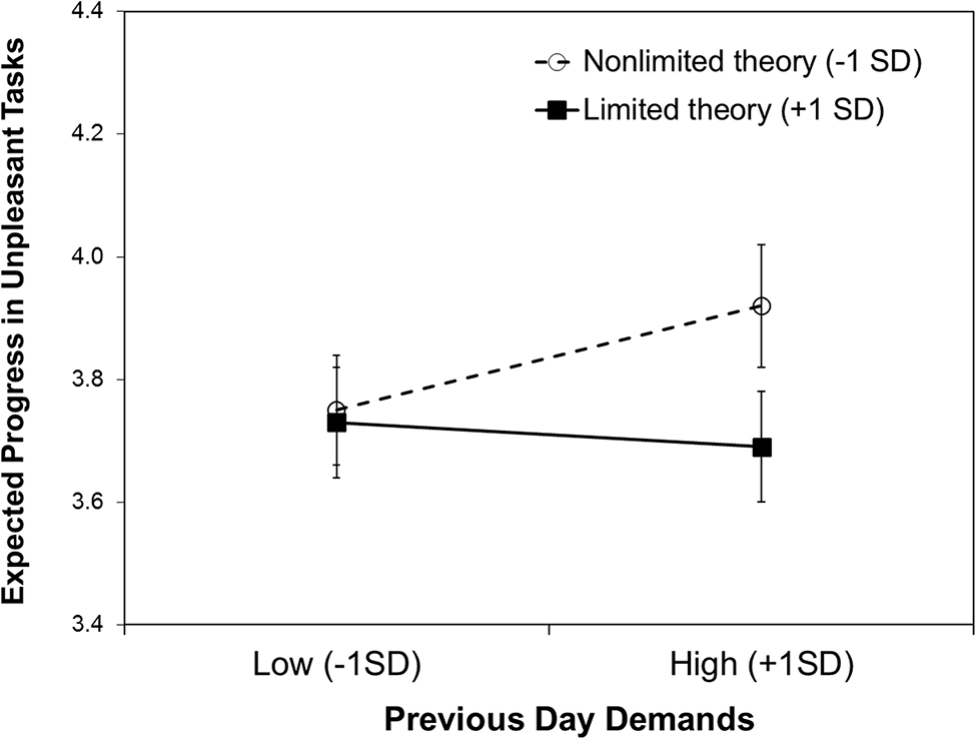
**Expected progress in unpleasant tasks predicted by willpower theory and previous day demands.** Error bars show ±1 SE.

Yes. We predicted the expected progress in unpleasant tasks with the same predictors as used in the previous model. The random effect of demands did not improve the model fit, *X*^2^(2) = 1.48, *p* = 0.476, and was therefore removed. As summarized in **Table 2**, there was no significant main effect of willpower theories, nor of previous day demands. However, the interaction between willpower theory and previous day demands was significant. The pattern of the interaction is depicted in **Figure 1**. We conducted simple slope analyses using a commonly used computational tool (Preacher et al., 2006), which probes 2-way interaction effects for hierarchical linear models and provides a *z*-test statistic for each slope at a conditional value of the moderator. The analysis showed that following a demanding day (+1 *SD*) there was a significant difference in expected progress between people with a limited and non-limited theory, *b* = -0.27, *SE* = 0.11, *z* = -2.44, *p* = 0.015. There was no difference following non-demanding days (-1 *SD*), *z* < 1, ns. People with a nonlimited theory (-1 *SD*) expected to make more progress after a demanding day than after a non-demanding day, *b* = 0.12, *SE* = 0.04, *z* = 2.82, *p* = 0.005, while people with a limited theory (+1 *SD*) did not differ in their expectations, *z* < 1, ns. We ran the same analysis for expected progress on pleasant tasks, but there were no main effects of willpower theory or previous day demands, and the interaction was also not significant, *t*s < 1.19, ns.

#### Do People with a Limited Theory Expect to be More Exhausted from Unpleasant Tasks, Particularly After a Demanding Day?

**FIGURE 2:**
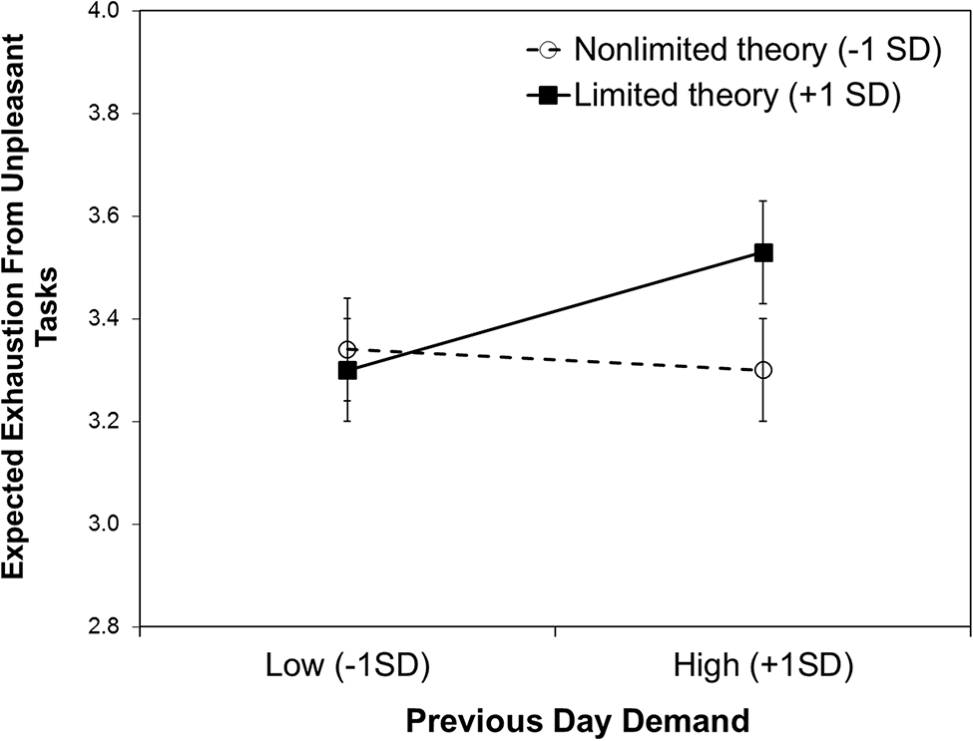
**Expected exhaustion from unpleasant tasks predicted by willpower theory and previous day demands.** Error bars show ±1 SE.

Yes. We predicted the expected exhaustion with the same variables as in the previous two models. As summarized in **Table 2**, there was no main effect of willpower theories or previous day demands. However, there was a significant interaction effect. The pattern of the interaction is depicted in **Figure 2**. Simple slope analyses showed that following a demanding day (+1 *SD*) people with a limited theory (+1 *SD*) expected to be more exhausted from unpleasant tasks than people with a non-limited theory (-1 *SD*), *b* = 0.29, *SE* = 0.11, *z* = 2.65, *p* = 0.008. There was no significant difference in expected exhaustion following non-demanding days (-1 *SD*), *z* < 1, ns. Further, people with a limited theory expected to be more exhausted from unpleasant tasks following a demanding day (+1 *SD*) than following a non-demanding day (-1 *SD*), *b* = 0.13, *SE* = 0.05, *z* = 2.83, *p* = 0.005. Among people with a non-limited theory, the expected exhaustion was low independent of previous day demands, *z* < 1, ns. The same model was used to predict expected exhaustion from pleasant tasks. Neither the main effects of willpower theories, nor previous day demands, or the interaction were significant, *t*s < 1.13, ns.

#### Are People with a Limited Theory Less Effective in Goal Striving, Particularly After a Demanding Day?

**FIGURE 3:**
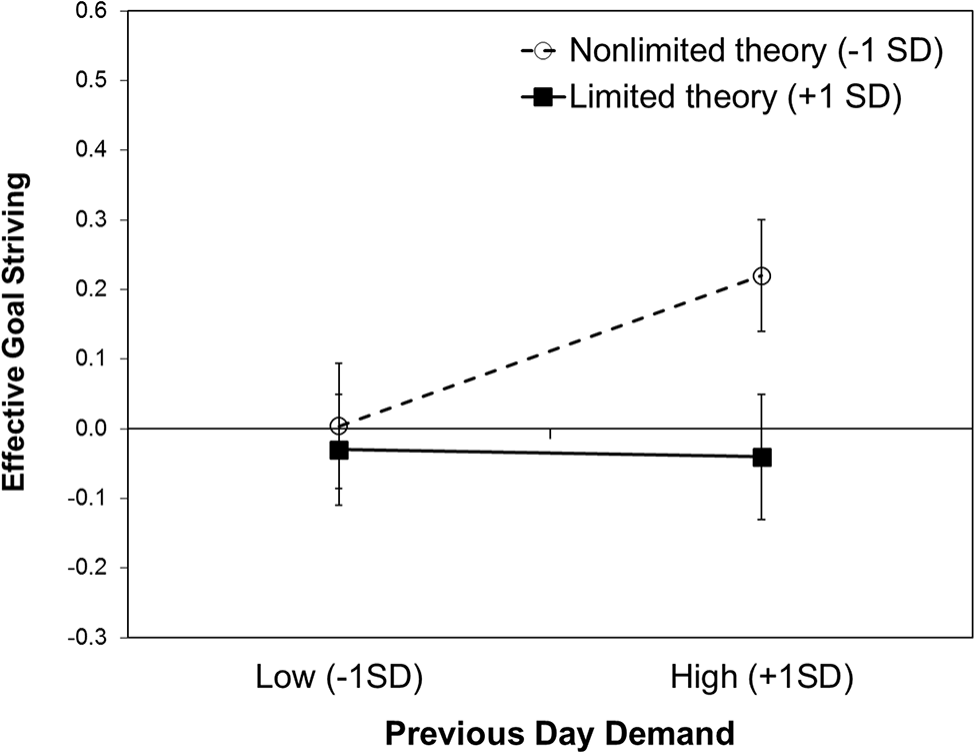
**Effective goal striving predicted by willpower theory and previous day demands.** Error bars show ±1 SE.

Yes. We predicted the overall effective goal striving on a day using the same variables as in the previous models. The results are summarized in **Table 3**. There was no main effect of willpower theory, but a significant effect of previous day demands. Following a demanding day, people were more productive. However, the effect was moderated by willpower theories. The pattern of the interaction is depicted in **Figure 3**. Simple slope analysis showed that after a demanding day (+1 *SD*) people with a non-limited theory (-1 *SD*) reported more effective goal striving than people with a limited theory (+1 *SD*), *b* = -0.32, *SE* = 0.13, *z* = -2.51, *p* = 0.012. After a non-demanding day, there was no difference in effective goal striving, *z* < 1, ns. People with a non-limited theory showed more effective goal striving following a demanding day than following a non-demanding day, *b* = 0.18, *SE* = 0.05, *z* = 3.34, *p* = 0.001. For people with a limited theory goal striving was overall less effective independent of previous day demands, *z* < 1, ns.

**Table 3:** Linear multilevel model predicting effective goal striving.

		Effective goal striving			
		
				95% CI		
Variable	*b*	*SE*	*p*	lower	upper
Intercept	-0.15	0.18	0.384	-0.50	0.19	
Day	-0.05	0.02	0.014	-0.10	-0.01	
Week	0.40	0.13	0.001	0.16	0.65	
Optimism	0.12	0.06	0.054	0.00	0.23	
Pessimism	0.02	0.06	0.710	-0.09	0.14	
Self-efficacy	0.20	0.11	0.076	-0.02	0.42	
Person mean demands	0.08	0.07	0.256	-0.06	0.22	
Same day demands	0.04	0.03	0.165	-0.02	0.10	
Previous day demands	0.07	0.03	0.018	0.01	0.13	
Willpower theory	-0.15	0.09	0.082	-0.32	0.02	
Willpower theory^∗^ Previous day demands	-0.14	0.06	0.014	-0.25	-0.03	
Variance components						
Intercept (*SD*)	0.36			0.30	0.44	
Residual (*SD*)	0.75			0.71	0.79	

*df_within_ = 147, df_between_ = 752.*

### Do Expectations Mediate the Effects of Willpower Theories and Previous Day Demands on Effective Goal Striving?

No. We tested whether the effect on effective goal striving was mediated by expectations and included expectations about the amount of unpleasant tasks, progress, and exhaustion (all person-mean centered) in the model. Effective goal striving was positively associated with the expected number of unpleasant tasks, *b* = 0.13, *SE* = 0.05, *t*(662) = 2.80, *p* = 0.005, and expectations of progress, *b* = 0.19, *SE* = 0.04, *t*(662) = 4.91, *p* < 0.001. Expected exhaustion from unpleasant tasks was not significantly related to goal striving, *b* = 0.04, *SE* = 0.04, *t*(662) = 1.11, *p* = 0.266. Although expectations explained some variance in goal striving the effect of the interaction between willpower theories and previous day demands remained significant when expectations were controlled, *b* = -0.12, *SE* = 0.06, *t*(662) = -2.05, *p* = 0.041. This finding suggests that expectations cannot account for the effect of willpower theories on effective goal striving.

## Discussion

Previous studies found that people with a limited theory show lower self-regulation with respect to a challenging goal when self-regulatory demands are high (Job et al., 2010). The aim of the present diary study was to examine whether willpower theories also moderate the effect of within-person changes in demands on effective goal striving. We found that people with a non-limited theory reported more effective goal striving than people with a limited theory when the previous day was demanding. Additionally, we examined the role of day-specific expectations (reported in the morning) and general expectations (i.e., optimism, pessimism, self-efficacy). We predicted that day-specific expectations mediate effects of willpower theories on effective goal striving (reported retrospectively in the evening). As expected, a limited theory predicted lower expectations regarding progress on unpleasant tasks (but not on pleasant tasks) and higher expected exhaustion from unpleasant tasks (but not on pleasant tasks) if the previous day was demanding. Although morning expectations were related to effective goal striving they did not mediate the effect of willpower theories and previous day demands. In sum, the findings replicate previous findings suggesting that willpower theories affect self-control when demands accumulate (Job et al., 2010, 2015b). Thereby, the present study also contributes to the current discussion in the self-control literature and supports alternative perspectives that provide motivational rather than resource-focused explanations for self-control failure (Inzlicht and Schmeichel, 2012; Kurzban et al., 2013; Inzlicht et al., 2014; Kool and Botvinick, 2014).

In the present study, we controlled for different variables at the day-level and the person-level to rule out alternative explanations. First, we controlled for persons’ mean level of demands and demands experienced on the day when participants reported their expectations and their effective goal striving. Further, previous day demands were person-mean centered. That means, previous day demands reflect the increment of the person experiencing more or less demands on a specific day than she would on average and controlling for the effects of being a person with generally high demands and the demands on the very same day. Thus, we can rule out that the effects of the previous day were due to people having more demands in general. Further, it is very unlikely that reports of effective goal striving were biased by people’s demands ratings, because the critical demand measure was completed 24 hours before the effective goal striving measure. Second, we controlled for people’s optimism, pessimism, and their level of self-efficacy to assure that willpower theories and not general expectations were driving the observed effects. Although, people with a non-limited theory were more optimistic, less pessimistic, and had higher self-efficacy, it was not the shared variance between these constructs that determined the impact of previous day demands on morning expectations and effective goal striving. Although, recent research suggests that state self-efficacy explains why self-control performance suffers from previous attempts to self-control (Chow et al., 2015), general self-efficacy seems not to account for the effects of willpower theories on everyday self-regulation. However, since the study has a correlational design, we cannot rule out all possible third variables that might account for the observed effects, such as for instance achievement motivation. This would only be possible with an experimental design were willpower theories are manipulated. There are promising findings from lab studies suggesting that willpower theories can be manipulated in the short term (Job et al., 2010; Miller et al., 2012; Vohs et al., 2013). However, longer term manipulation of willpower theories in the field is still a challenge that remains for future research.

Previous field studies showed that particularly within times of high demands, people’s willpower theory predicts how successful they strive for their personal goals (Job et al., 2010). In the current study we replicated these findings with within-persons variations in daily demands. However, the pattern of the interaction suggests a positive effect of demands for people with a non-limited theory rather than a negative effect of demands for people with a limited theory. This “energizing” pattern might be explained by the fact that atypically people in the present sample agreed more with a non-limited theory than with a limited theory (Job et al., 2010, 2015a). The greater endorsement of a non-limited theory in the present sample might also explain why previous day demands were overall positively related to effective goal striving on the next day and not negatively. As mentioned earlier, a non-limited theory includes the idea that engaging in strenuous tasks can activate one’s willpower and therefore improve subsequent performance.

The finding that previous day demands in general were related to more effective goal striving on the following day is surprising, because previous research suggests that demands lead to depletion-like effects causing less effective goal striving (Baumeister and Heatherton, 1996; Oaten and Cheng, 2005). The fact that demands can have positive and negative effects on performance is also reflected in new models of stress. Although the classic view on stress is that it undermines performance (e.g., Glass and Singer, 1972), more recent models propose different types of stress, which have different effects on performance. For instance, the Challenge-Hindrance-Modell (Cavanaugh et al., 2000) suggests that stressors can be categorized either as *challenge stressors* which promote performance (e.g., time pressure) or *hindrance stressors* which harm performance (e.g., red tape). Studies often measure these different types of stressors, which are categorized a priori as challenge or hindrance stressors. However, it has been argued that the pre-categorization of stressors ignores the socio-cognitive approaches to stress, which conceptualize stress as a function of a person’s appraisals (Edwards et al., 2014). The effects of stressors on performance might depend on the individual’s appraisal of the specific stressor as threat or challenge. If a person perceives to have sufficient resources to cope with the demands in his or her environment stressors might be perceived as a challenge and promote rather than impair performance (Edwards et al., 2014). The present study suggests that willpower theories determine the impact of previous day demands on next day’s self-regulatory performance. The appraisal of self-regulatory demands as a challenge or hindrance might play an important role in this context. When people believe that their willpower is limited they might perceive to have not sufficient resources to overcome demands on the next day. Future studies should therefore investigate whether self-regulatory demands are perceived differently when people endorse a limited or non-limited theory.

An open question that remains from the present research concerns the role of expectations. Although morning expectations were related to effective goal striving the effect of willpower theories and previous day demands remained significant when expectations were controlled. One reason for this finding might be that the measure of expectations did not exactly match the measure for effective goal striving. Perhaps, the proposed mediation would emerge if measures of expectations and effective striving would both refer to the same goal or unpleasant task. Future studies should therefore ask people in the morning about the specific unpleasant task or goal for that day and then assess their progress in that specific task or goal in the evening. Due to this methodological limitation of the study, we cannot rule out that expectations play a role in explaining effects of willpower theories on different outcomes.

Recently, studies suggest that one part of the mechanism might be different goals people strive for after they exerted self-control (Job et al., 2015a). Following a self-control task, people with a limited theory have the goal to rest and are more likely to engage in resting behavior (Job et al., 2015a). However, the pursuit of the goal to rest is not incompatible with different expectations about future self-control performance. These two mechanisms might go hand in hand. People with a limited theory might expect to perform less well on future tasks because they believe that their resources are depleted. Further, they might pursue the goal to rest and replenish these resources in order to increase their likelihood of performing well on the upcoming task. Future field studies should assess both expectations and the goal to rest following a demanding day.

We also want to mention some limitations of the study with regard to the sample and the measures used. Because our main interest was to test a conceptual hypothesis that willpower theories affect goal striving and expectations in interaction with previous demands we chose a homogenous student sample for this study. The question, however, remains whether the findings can be generalized to the general population and whether they are affected by different variables such as age, culture, or social class. Further, the use of self-report measures is a limitation of the present study. Sampling behavioral data on effective goal striving would be a great improvement for future studies.

## Conclusion

The present study supports James’ (1907) notion that daily fluctuations in levels of energy might depend on people’s motivation to exert their mental and physical resources. The present findings suggest that beliefs about willpower determine whether demands prompt people to save their energies and put their goals on hold or whether they encourage them to lean in and fully tap into their resources.

## Author contributions

KB was involved in the conception and design of study, as well as the collection, analysis, and interpretation of data. Further, KB was involved in drafting the work and gave final approval of the version to be published. KB gives agreement to be accountable for all aspects of the work in ensuring that questions related to the accuracy or integrity of any part of the work are appropriately investigated and resolved.

VJ was involved in the conception and design of study, as well as the interpretation of data. Further, VJ was involved in drafting and revising the work and gave final approval of the version to be published. VJ gives agreement to be accountable for all aspects of the work in ensuring that questions related to the accuracy or integrity of any part of the work are appropriately investigated and resolved.

## Conflict of Interest Statement

The authors declare that the research was conducted in the absence of any commercial or financial relationships that could be construed as a potential conflict of interest.
